# A Novel LncRNA, AC091729.7 Promotes Sinonasal Squamous Cell Carcinomas Proliferation and Invasion Through Binding SRSF2

**DOI:** 10.3389/fonc.2019.01575

**Published:** 2020-01-24

**Authors:** Boyu Yu, Linmei Qu, Tianyi Wu, Bingrui Yan, Xuan Kan, Xuehui Zhao, Like Yang, Yushan Li, Ming Liu, Linli Tian, Yanan Sun, Qiuying Li

**Affiliations:** ^1^Department of Otorhinolaryngology, Head and Neck Surgery, The Second Affiliated Hospital, Harbin Medical University, Harbin, China; ^2^Department of Otorhinolaryngology, Head and Neck Surgery, The Fifth Affiliated Hospital, Harbin Medical University, Daqing, China; ^3^Department of Otorhinolaryngology, Head and Neck Surgery, Henan Provincial People's Hospital, People's Hospital of Zhengzhou University, Zhengzhou, China

**Keywords:** long non-coding RNA, AC091729.7, sinonasal squamous cell carcinomas, serine/arginine rich splicing factor 2, prognosis

## Abstract

Long non-coding RNAs (lncRNAs) play important roles in various biological progresses of carcinogenesis. However, the function of lncRNAs in human sinonasal squamous cell carcinoma (SNSCC) remains greatly unclear. In the current study, lncRNA AC091729.7 expression was examined in SNSCC samples by using microarray, RNA *in situ* hybridization (ISH) and real-time fluorescence quantitative PCR (qRT-PCR). Cell viability, colony-formation, wound-healing, and transwell assays were applied to SNSCC cells. Xenograft mouse models were employed to evaluate the role of AC091729.7 in growth of SNSCC *in vivo*. Human protein microarray (Huprot^TM^ Protoarray) and RNA immunoprecipitation (RIP) were used for identifying AC091729.7 binding proteins in SNSCC. Results showed AC091729.7 was upregulated and closely connected with the survival of the SNSCC patients. Knockdown of AC091729.7 suppressed SNSCC cell migration, proliferation, invasion *in vitro*. Furthermore, downregulation of AC091729.7 could inhibit the growth of SNSCC *in vivo*. Moreover, Human protein microarray and RIP suggested that AC091729.7 directly combine with the serine/arginine rich splicing factor 2 (SRSF2). Our results suggest that in the cell progression of SNSCC, lncRNA AC091729.7 plays a carcinogenic role and serves as a novel biomarker and latent curative target in SNSCC patients.

## Introduction

Nasal cavity and paranasal sinus malignancies account for 3% of those in the head and neck region and 1% of the total (1). Squamous cell carcinoma (SCC) is the most common histological type of nasal malignancies (2). Sinonasal squamous cell carcinomas (SNSCC) tend to occur in advanced stages, accompanied by local damage and unfavorable prognosis. The present therapeutic methods include operation. However, radiation therapy, as well as chemotherapy, might be imperative for some patients (3–7). Despite improvements in surgery and radiotherapy, the prognosis of these tumor patients remains poor, with a 5-year survival rate of about 40% and local recurrence as the leading cause of death (3–5). Long non-coding RNAs (lncRNAs) are a class of non-coding transcripts >200 nucleotides in length. LncRNAs can be used as potential prognostic biomarkers for a variety of tumors (8–10). Recent studies suggested that lncRNAs influence the progression of cancer (11, 12). For instance, HOTAIR, MEG3, MALAT-1, H19, and GAPLINC may play a part in carcinogenesis (13–17). A number of studies have showed diagnostic and prognostic values of lncRNAs in in head and neck squamous cell carcinoma. Such as long non-coding MIR205HG could cause unlimited proliferation of head and neck squamous cell carcinoma (18). Long Non-coding RNA FAM225A promotes Nasopharyngeal Carcinoma tumorigenesis and metastasis (19). LncRNA SSTR5-AS1 promotes progression and metastasis of laryngeal squamous cell carcinoma (20). LncRNA-p23154 promotes the invasion-metastasis potential of oral squamous cell carcinoma (21). However, the functions and the mechanisms underlying lncRNA in SNSCC have not been reported previously. Thus, to explore the role of lncRNAs in SNSCC, we assessed the expression of lncRNA AC091729.7; it was found to be associated with the clinicopathological characteristics in SNSCC patients. Our results showed that AC091729.7 is overexpressed in SNSCC tissues, and knockdown of AC091729.7 can inhibit the proliferation of SNSCC cells both *in vitro* and *in vivo*. Herein, we firstly demonstrated that AC091729.7 binds to SRSF2. Also, a novel mechanism showed that lncRNA regulates the pathogenesis of SNSCC.

## Materials and Methods

### Samples

A total of 29 patients who had sinonasal cancer and underwent SNSCC surgical resection at the Department of Otorhinolaryngology of the Second Affiliated Hospital of Harbin Medical University between October 2009 and July 2013 were recruited in this study. Profile analysis was performed in five patients, and qRT-PCR validation was performed in 24 patients. After surgical resection, fresh paired cancerous and adjacent normal tissues were collected and frozen in liquid nitrogen.

Another 60 paraffin samples of SNSCC paired cancerous and adjacent normal tissues were collected from the Department of Pathology of the Second Affiliated Hospital of Harbin Medical University. All patients provided written informed consent by the Helsinki Declaration. The Ethics Committee of Harbin Medical University approved this study protocol.

### Microarray Analysis

Total RNA was extracted from SNSCC and homologous adjacent non-neoplastic tissues using TRIzol reagent (Invitrogen, USA) and quantified by NanoDrop 1000. The integrity of the RNA was measured by standard denatured agarose gel electrophoresis. The human 8 × 60 K lncRNA array was produced by Arraystar Company (USA). Over 25,000 lncRNAs were gathered from the canonical data sources encompassing UCSC, NCBI RefSeq, RNAdb, and NRED. Approximately 5 μg of total RNA was used for tag and array hybridization from each sample.

### qRT-PCR

Purified total RNA from SNSCC tissues or RPMI-2650 cells was used to test the expression of lncRNA AC091729.7. cDNA was synthesized using the Reverse Transcription Kit (Takara, China). qRT-PCR was performed using the SYBR Green Master Mix (Roche, Switzerland). Primers were as follows: AC091729.7 forward: 5′- GGCGAGGTGATTCACAGTGGAG-3′, and reverse: 5′-CAGCGGTCACGGAGCAGTTG-3′; GAPDH forward: 5′- GGGAGCCAAAAGGGTCAT-3′reverse: 5′-GAGTCCFTTCCACGATACCAA-3′; SRSF2 mRNA: forward: 5′-CCACTCAGAGCTATGAGCTACG-3′; reverse: 5′-ACTCCTTGGTGTAGCGATCC-3′.

### ISH

The expression of AC091729.7 was examined in 60 paraffin samples of SNSCC and adjacent non-cancerous specimens by ISH employing the RNAscope technology (Brown, USA). AC091729.7 target probe, along with the positive and negative control probes, was provided by Advanced Cell Diagnostics (ACD). A common housekeeping protein, peptidyl-prolyl isomerase B (PPIB), was used as the positive controls probe, while the negative control probe of the bacterial protein dihydrodipicolinate reductase (DapB) was utilized. Brown punctate staining was observed in the cytoplasm or nucleus, indicating a positive test. The expression levels were divided into five grades according to the manufacturer's rating criteria. The slides were scored at 200× magnification using the RNAscope scoring guide: 0: unstained; 1: each tumor cell had 1–3 spots; 2: each tumor cell had 4–10 spots; 3: each tumor cell had >10 spots and <10% of the tumor cells had clusters of spots; 4: each tumor cell had >10 spots, and >10% of the tumor cells had clusters of spots. The low expression of AC091729.7 is indicated by 0 and 1 scores, while the high expression is represented by 2, 3, and 4 scores.

### Subcellular Fractionation Location

Nuclear and cytoplasmic components were isolated using the PARIS Kit (Life Technologies, USA) based on the manufacturer's instructions.

### Cell Transfection

The human RPMI-2650 (nasal squamous cell carcinoma) cell line was provided by ATCC (Bethesda, USA). The cells were grown in MEM supplemented with 10% fetal bovine serum (FBS), 1% NEAA, and L-glutamine at 37°C under 5% CO_2_.

The shRNA lentiviral transfer vector and the lentiviral control vector (GFP-lentivirus) were synthesized by Genechem (Shanghai, China). The sequence was as follows: GGAAATGCTTTGTGTACTT. The pcDNA3.1 vector (control vector), lncRNA AC091729.7 overexpression vector (pcRNA), and the siRNA targeting SRSF2 or non-specific scrambled control siRNA (Silencer Negative Control) were provided by Genechem. For overexpression assay, the cells were allowed to grow for 24 h prior to transfection with pcDNA3.1 or pcDNA3.1-SRSF2 (pcSRSF2). The transfected cells were used in subsequent experiments.

### Cell Counting Kit-8 (CCK-8)

The CCK-8 assay was used to detect the effect of lncRNA AC091729.7 on the proliferation of cells. Cells were seeded in the 96- well plate at a density of 2 × 10^3^/well and incubated for 24, 48, 72, and 96 h, respectively. A volume of 10 μL CCK-8 reagent was added to each well at the specific time point, and the plate was incubated for an additional 4 h. The absorbance was measured at 450 nm using the Microplate reader (Bio-Rad, Richmond, CA, USA).

### Colony Formation Assay

Approximately, 500 cells were inoculated in each well of the 6-well plate and incubated for about 2 weeks at 37°C in 5% CO_2_; the clone formation could be observed by the naked eye. The colonies were fixed with 4% paraformaldehyde for 15 min and stained with 0.1% crystal violet for 15 min. The number of clones was counted under a microscope.

### Xenografts in Mice

Twelve BALB/c nude mice, 5–6 week-old were purchased from Beijing Vital River Laboratory Animal Technology Co. Ltd and raised in a sterile environment at constant temperature and humidity according to the standard guidelines by Harbin Medical University. An equivalent of 1 × 10^6^ (100 μL) RPMI-2650 cells were injected subcutaneously in the dorsal scapula region of all mice. When tumors grew to about 0.5–0.6 cm^3^ in size, we injected into the tumor weekly for 3 weeks. The experimental group (*n* = 6) received an injection of 100 μL AC091729.7 shRNA lentivirus, while the control group (*n* = 6) received an injection of 100 μL control lentivirus. Tumors were harvested at 1 week after the end of treatment.

### Wound Healing Assay

When the cells were at 80–90% confluent, the monolayer was scratched using sterile 200-μL pipette tips. The wound was photographed under a microscope at 0 and 48 h, and then estimated the migration distance of the cells using soft.

### Transwell Invasion Assay

The invasion capacity of the cells was measured by a Matrigel invasion chamber. In serum-free media, a total of 1 × 10^5^ cells were inoculated in the upper chamber of each insert (24-well plates, 8-mm pore size, Corning). The culture medium containing 20% FBS was placed in the lower chamber. After 48 h, the rest of the cells on the upper surface were wiped away with a cotton swab. The cells that invaded from the upper surface were fixed with 4% paraformaldehyde and stained with 5% crystal violet. Subsequently, five fields of view were randomly selected microscopically to observe and count the number of invasive cells.

### Protoarray and RIP

In the Huprot^™^ Protoarray hybridization, all oligonucleotide sequences were labeled with fluorescence. The data were provided by H-WAYEN (Shanghai, China). Magna RIP RNA-Binding Protein Immunoprecipitation Kit (Millipore, USA) was used to perform the RIP experiment following the manufacturer's instructions.

### Western Blotting

RPMI-2650 cells were lysed with RIPA lysis buffer (Beyotime, China) containing the protease inhibitor cocktail. Protein samples were resolved by SDS-PAGE and transferred to a PVDF membrane. Then, the membrane was blocked with Blocking Buffer (Beyotime) and probed with primary anti-SRSF2 antibody (Santa Cruz Biotechnology, France). GAPDH was used as an internal control on the same membrane. Next, the membrane was incubated with the horseradish peroxidase-conjugated (HRP) secondary antibody for 1 h. The immunoreactive signal was processed visually by the ECL detection system (Beyotime).

### Statistical Analysis

Statistical Package SPSS 17.0 was used for all statistical analyses. Two samples were compared and analyzed by Student's *t*-test. The correlation between AC091729.7 and the clinicopathological features was assessed by *X*^2^ test. The diagnostic value of AC091729.7 expression in SNSCC was evaluated by analysising the receiver operating characteristic (ROC) curve. Kaplan–Meier survival curve was used to calculate the overall survival of SNSCC patients. *P* <*0.05 was considered as statistical significance*.

## Results

### LncRNA AC091729.7 Is Up-Regulated and Related to Clinicopathological Features and Overall Survival of SNSCC Patients

**Figure 1:**
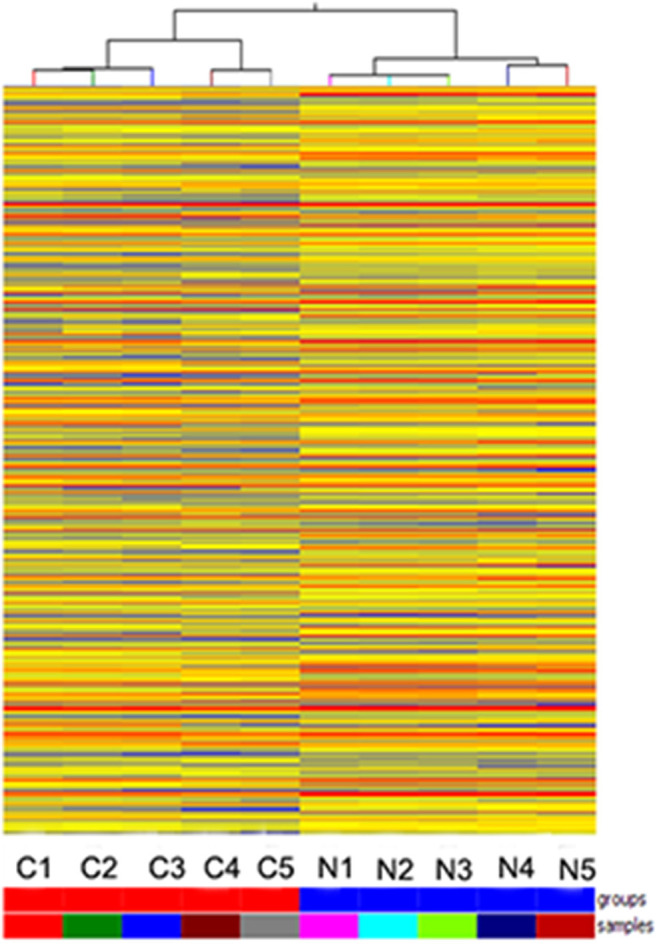
Hierarchical clustering is shown as a heat map, and 1,066 differently expressed lncRNA levels are shown in color scales (Red indicates high relative expression, and blue indicates low relative expression). Columns C1–C5 are five different SNSCC samples, and columns N1–N5 represent the corresponding non-cancerous tissues.

Hybridization microarray images of SNSCC tissues and non-neoplastic tissues identified 21909 lncRNAs. Microarray data analysis showed 1,066 differently expressed lncRNAs between SNSCC and non-neoplastic tissues, of which 393 were up-regulated and 673 were down-regulated (Figure 1). Herein, AC091729.7 (fold-change 5.014; *p* = 0.001247) was found to have the significant fold-change.

**Figure 2:**
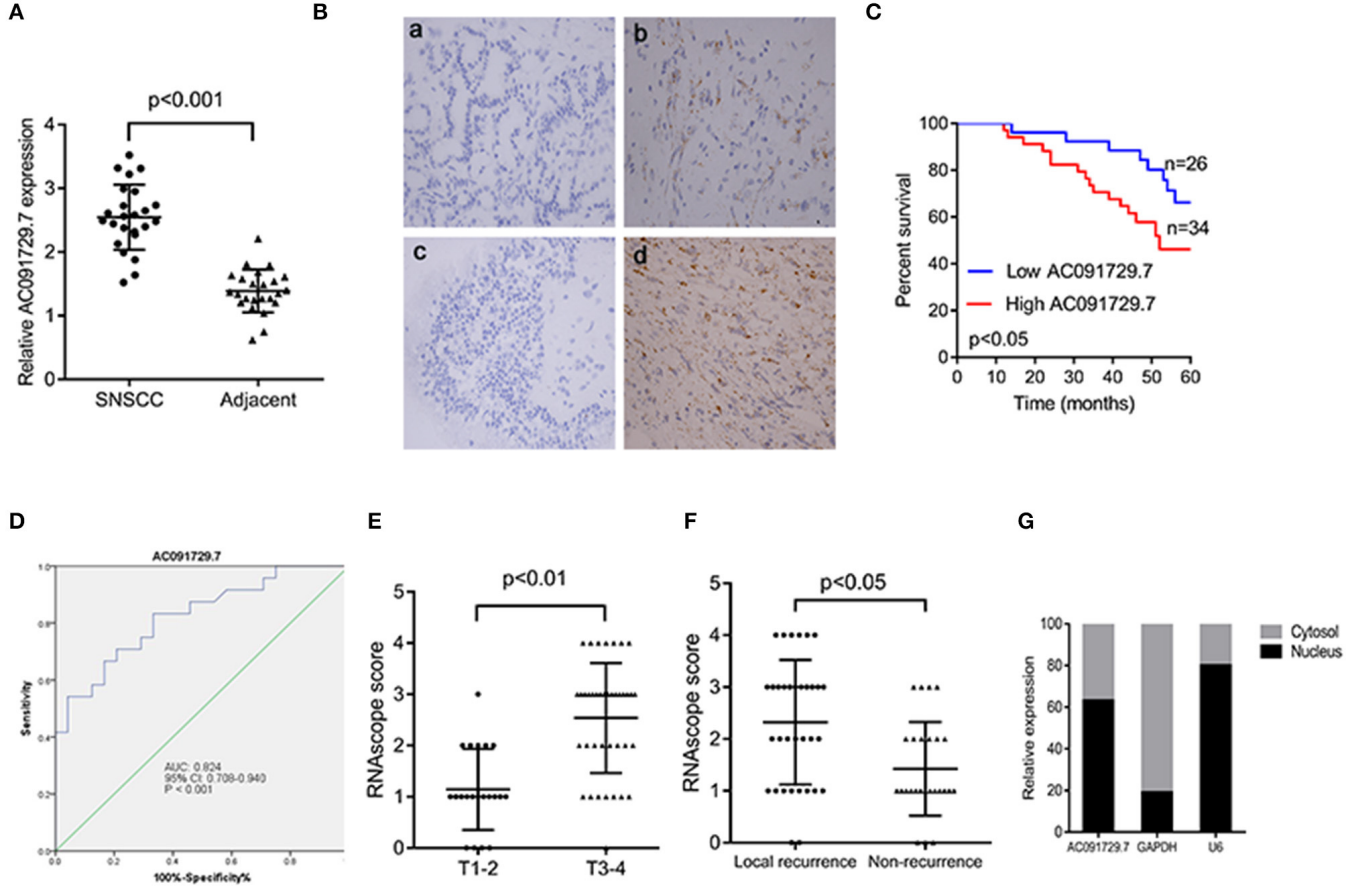
AC091729.7 is overexpressed in SNSCC tissues. **(A)** Quantitative analysis of 24 paired SNSCC tissues by qRT-PCR. **(B)** Representative ISH images of AC091729.7 expression in SNSCC. (a) Adjacent tissue; (b) SNSCC tissue; (c) Negative control; (d) Positive control. **(C)** The overall survival in 60 SNSCC patients is represented by Kaplan–Meier curves. High AC091729.7 expression correlates with poor prognosis in SNSCC patients. **(D)** The area under ROC curve of AC091729.7 in distinguishing SNSCC tissues and normal tissues was 0.824. **(E)** AC091729.7 is correlated with T classification. **(F)** AC091729.7 is correlated with recurrence. **(G)** The level of AC091729.7 in nucleus or cytoplasm. RNA was extracted from the nuclear and cytoplasmic fractions of RPMI-2650 cells, and the AC091729.7 expression in the nuclear and the cytoplasmic fraction was measured by qRT-PCR. GAPDH was used as a cytosolic marker, and U6 was used as a nuclear marker.

In order to explore the expression of AC091729.7 in SNSCC tissues, we first determined the expression of AC091729.7 in 24 fresh paired tissues of SNSCC by qRT-PCR; high expression was found in SNSCC tissues as compared to the non-cancerous tissues (Figure 2A). Consistently, ISH results showed that the level of AC091729.7 was also significantly increased in 60 SNSCC tissues as compared to that in the corresponding adjacent tissues (Figure 2B). The correlation between AC091729.7 expression and the overall survival of patients was analyzed by the Kaplan–Meier method analysis (log-rank test). As shown in Figure 2C, the overall survival time of 34 patients with high AC091729.7 expression was significantly shorter than the 26 patients with low AC091729.7 expression (*p* < 0.05). The ROC curves showed that the SNSCC tissues was obviously separated from the adjacent normal tissues, with an area under the curve of 0.824 (95% confidence interval, 0.708–0.940; Figure 2D). Furthermore, we evaluated the correlation between AC091729.7 expression and the clinicopathological parameters in 60 SNSCC patients. As presented in Table 1, the expression of AC091729.7 was significantly correlated with T classification (Figure 2E, *p* = 0.002) and local recurrence (Figure 2F, *p* = 0.018); however, it was not significantly related with sex, age, smoking status, and N classification (*p* > 0.05). Next, the percentage of AC091729.7 expression in the cytoplasmic and nuclear fractions of RPMI-2650 cells was determined and was found to be primarily localized in the nucleus in RPMI-2650 cells (Figure 2G).

**Table 1:** Correlation between AC091729.7 expression and clinicopathological features in SNSCC.

	**AC091729.7**		
**Parameters (*n* = 60)**	**Low (*n* = 26)**	**High (*n* = 34)**	***P***
Sex			1.000
Male (18)	8	10	
Female (42)	18	24	
Age			0.065
≤60 (38)	20	18	
>60 (22)	6	16	
Smoking status			0.596
Smoker (36)	17	19	
Non-smoker (24)	9	15	
T classification			0.002^*^
T1-2 (21)	15	6	
T3-4 (39)	11	28	
N classification			0.526
N0 (48)	22	26	
N1 (12)	4	8	
Local recurrence			0.018^*^
Yes (34)	10	24	
No (26)	16	10	
Distant metastasis			0.320
Yes (11)	3	8	
No (49)	23	26	

* *p < 0.05*.

### Knockdown of AC091729.7 Inhibits SNSCC Cell Proliferation and Invasion

**Figure 3:**
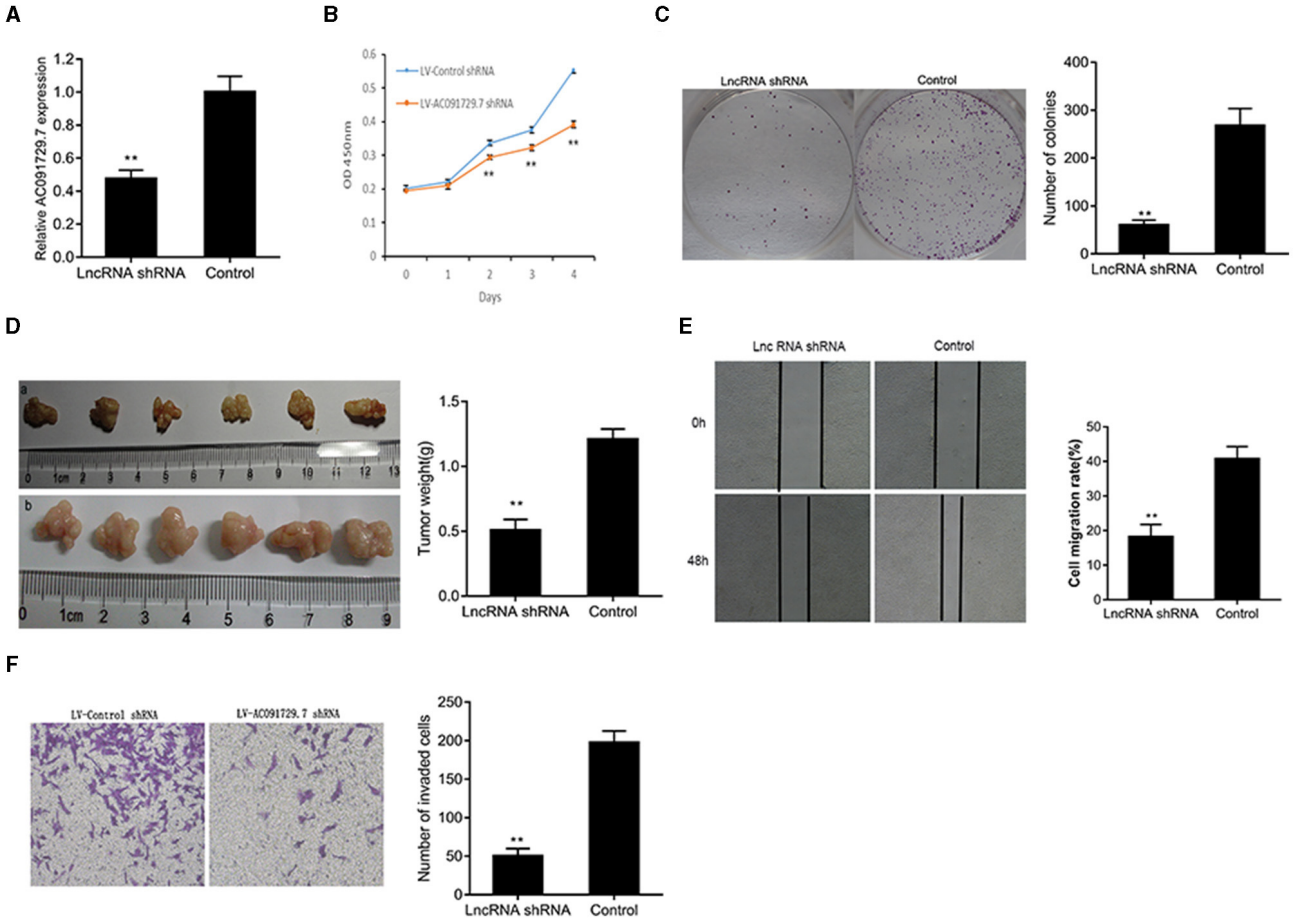
The knockdown of AC091729.7 inhibits the proliferation, migration, and invasion of SNSCC cells. **(A)** Relative RNA level of AC091729.7 was decreased in SNSCC cells with AC091729.7 knockdown. **(B)** CCK-8 showed that the viability of SNSCC cells was inhibited after the downregulation of AC091729.7 expression. **(C)** Colony-formation assay suggested that SNSCC cell proliferation was inhibited after AC091729.7 knockdown. **(D)** Subcutaneous xenograft SNSCC tumors developed in nude mice after RPMI-2650 cells were transfected with lentivirus encoding (a) AC091729.7 shRNA; (b) control shRNA. **(E)** Wound healing cell migration assay. **(F)** Transwell invasion assay (**p < 0.01).

To investigate the function of AC091729.7 on the proliferation of SNSCC cells, RPMI-2650 cells were satisfactorily transfected with sh-AC091729.7 using shRNA as a negative control for about 48 h (Figure 3A). As shown in Figure 3B, after sh-AC091729.7 transfection, the viability of RPMI-2650 cells decreased significantly at different time points (48, 72, and 96 h, respectively). Moreover, the colony assay further confirmed that the downregulation of AC091729.7 contributed to the repression of colony formation in SNSCC cells (Figure 3C). These results indicated that the downregulation of AC091729.7 suppresses the capacity of SNSCC *in vitro*. Furthermore, we used a mouse xenograft model to study the carcinogenic effect of AC091729.7 on the development of SNSCC *in vivo*. In the current study, all mice developed detectable tumors after subcutaneous injection of the cells. Compared to the control, AC091729.7 shRNA significantly inhibited the tumor growth in mice, and the tumor mass weight also reduced significantly (Figure 3D). The effects of AC091729.7 on the migration and invasion of RPMI-2650 cells were determined by wound healing and transwell assays. As shown in Figures 3E,F, after AC091729.7 downregulation, both migratory and invasive capabilities of RPMI-2650 cells were remarkably inhibited, thereby indicating that AC091729.7 promotes the migration and invasion of SNSCC cells.

### Overexpression of AC091729.7 Promotes SNSCC Cells Proliferation and Invasion

**Figure 4:**
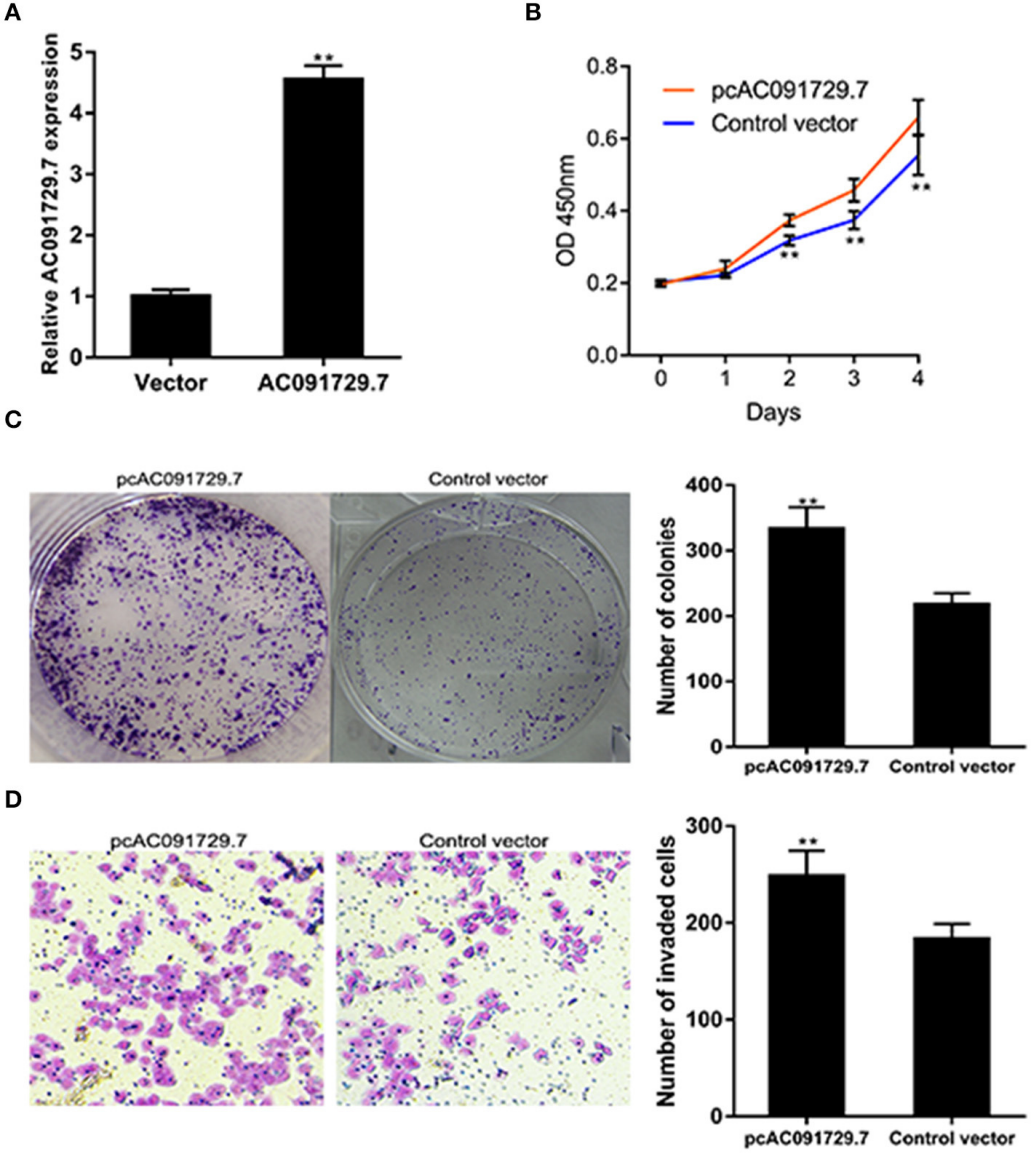
AC091729.7 overexpression influences SNSCC cell proliferation and invasion. **(A)** The relative RNA level of AC091729.7 was increased in SNSCC cells with overexpression of AC091729.7. **(B)** CCK-8 showed that the activity of SNSCC cells increased after overexpression of AC091729.7. **(C)** Colony-formation assay **(D)** Transwell invasion assay (**p < 0.01).

To further investigate the function of AC091729.7 on the proliferation of SNSCC cells, RPMI-2650 cells were satisfactorily transfected with pcAC091729.7 using vector as a negative control for about 48 h (Figure 4A). As shown in Figure 4B, after pcAC091729.7 transfection, the viability of RPMI-2650 cells increased significantly at different time points (48, 72, and 96 h, respectively). Moreover, the colony assay further confirmed that the overexpression of AC091729.7 contributed to enhanced colony formation and invasive capabilities in SNSCC cells (Figures 4C,D).

### SRSF2 Is Identified as a Potential Downstream Target of AC091729.7 in SNSCC Cells

**Figure 5:**
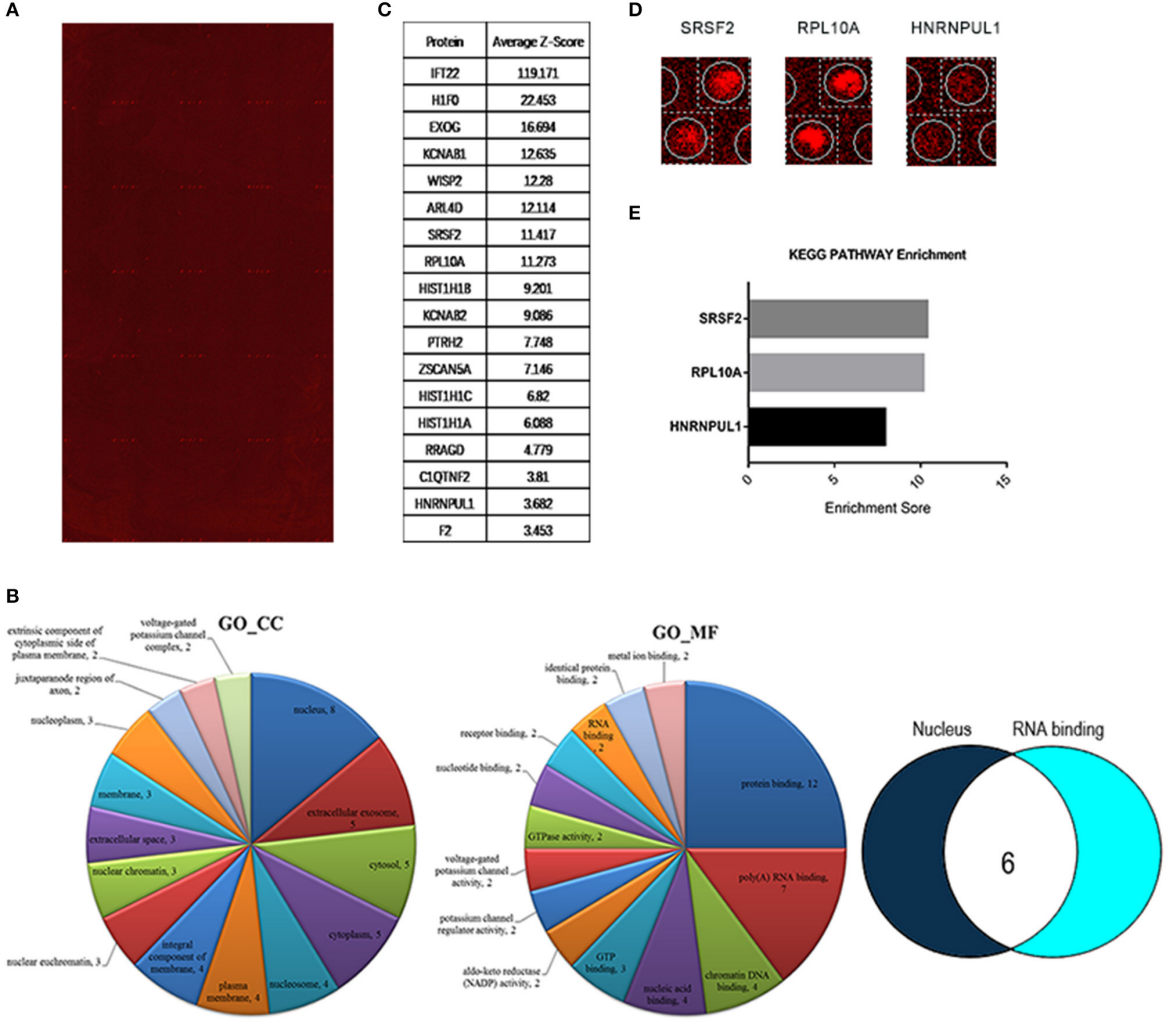
HuProt^™^ protoarray analysis of lncRNA AC091729.7 binding proteins. **(A)** HuProt^™^ human protoarray scan. This protoarray encompasses 20240 human full-length proteins, which is scan map of the RNA fragment consisting of the full-length sequence of lncRNA AC091729.7 hybridization. **(B)** Gene ontology (GO) analysis. The detected lncRNA AC091729.7 bound to nucleoproteins was selected for GO analysis. AC091729.7 was primarily expressed in the nucleus. After hybridization, the binding proteins were screened, and the nuclear and RNA-binding proteins were selected for further analysis. Intersection analysis identified six AC091729.7-binding proteins. **(C)** Table representing the *Z*-score of binding proteins. **(D)** Magnified protoarray scan signal. **(E)** KEGG (Kyoto Encyclopedia of Genes and Genomes) analysis. The detected three lncRNA AC091729.7-binding nucleoproteins were chosen for KEGG analysis. SRSF2 putatively bound to AC091729.7.

**Figure 6:**
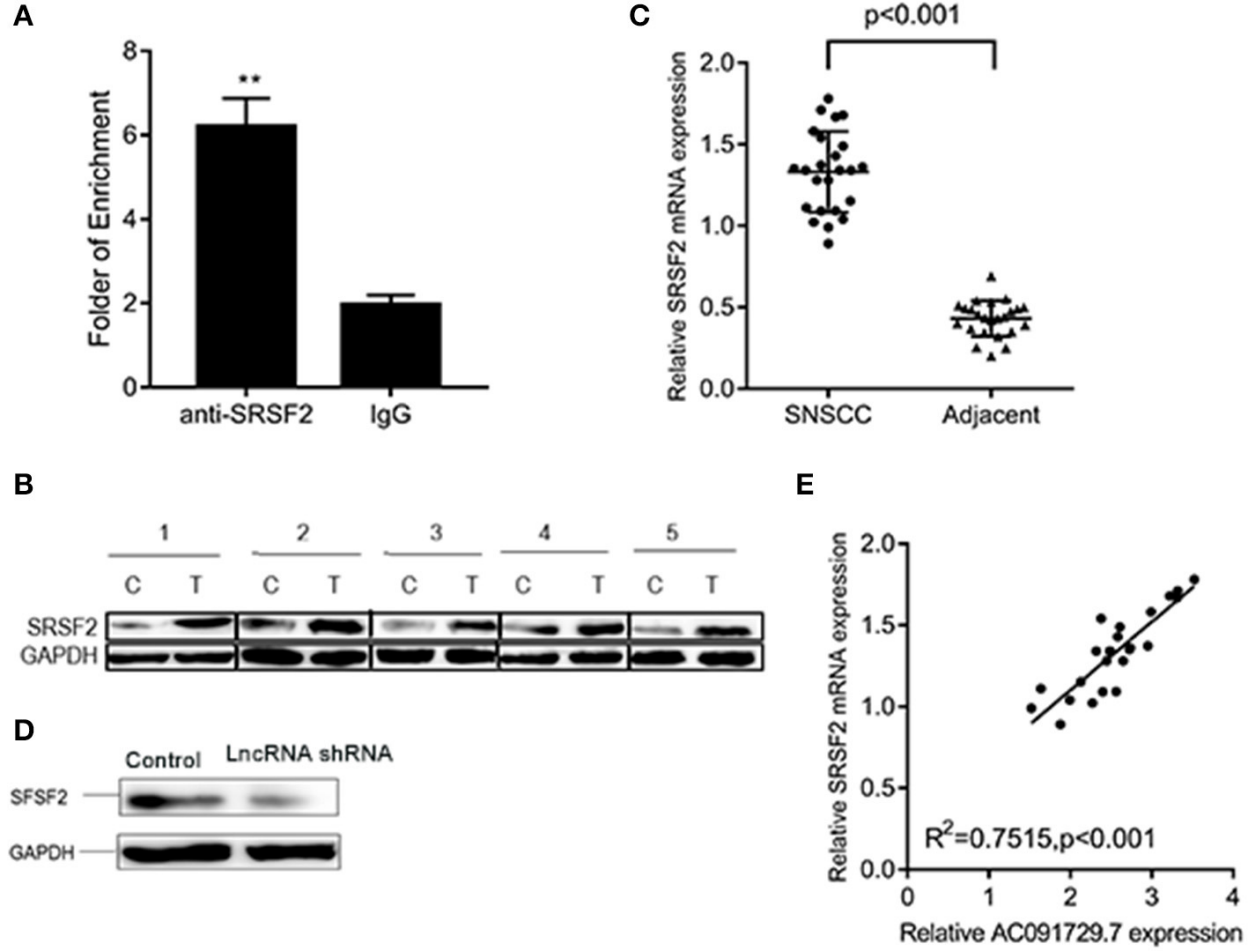
AC091729.7 interacted with SRSF2. **(A)** RIP assays revealed that AC091729.7 interacts with SRSF2 in SNSCC cells. IgG was used as a negative control. **(B)** Western blot results suggested that the level of SRSF2 protein was increased in SNSCC as compared to the adjacent tissues. **(C)** Quantitative analysis of 24 paired SNSCC tissues by qRT-PCR showed that *SRSF2* mRNA is overexpressed in cancer tissues. **(D)** Western blot showed that the level of SRSF2 was downregulated after AC091729.7 knockdown. **(E)** A positive correlation was established between SRSF2 mRNA expression and AC091729.7 level in the SNSCC tissues (**p < 0.01).

**Figure 7:**
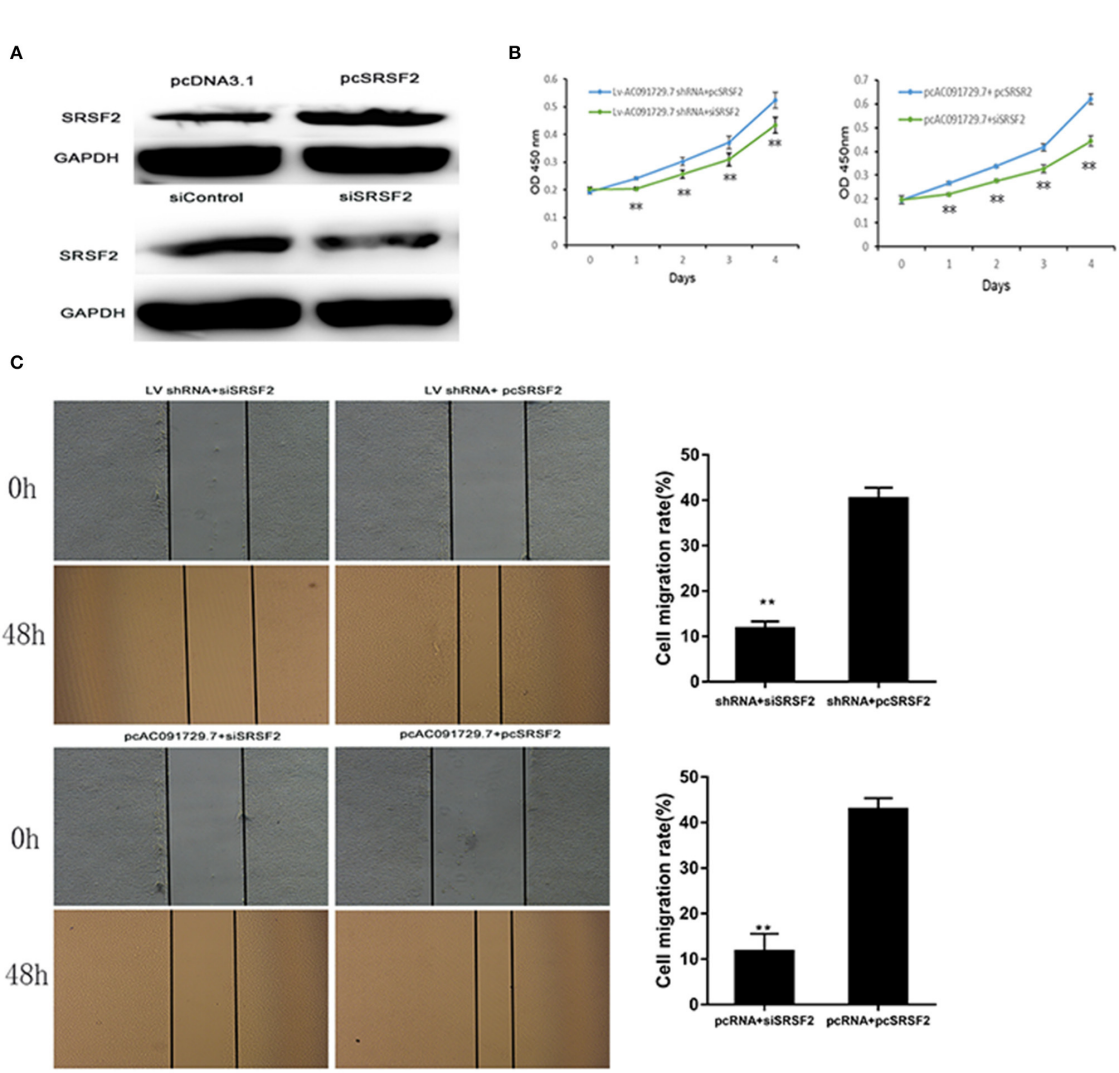
AC091729.7 promotes the migration and proliferation of SNSCC cells through the regulation of SRSR2 expression. **(A)** Silencing and overexpression of SRSF2 in SNSCC cells are illustrated by Western blot. The rescue experiment with CCK-8 **(B)** and wound healing **(C)** were performed in RPMI-2650 cells co-transfected with Lv AC091729.7 shRNA or pcAC091729.7 and siSRSF2 or pcSRSF2 (**p < 0.01).

In order to understand the potential mechanism of AC091729.7 function, we first interrogated a human proteome microarray (Figure 5). Results showed that AC091729.7 could potentially bind 18 proteins. Herein, we selected SRSF2, a 35-kDa serine/arginine-rich protein, as the subsequent study molecule owing to its high confidence protein-binding partners for AC091729.7. To further confirm the interaction between AC091729.7 and SRSF2, we performed RIP with an antibody against SRSF2. The SRSF2 antibody worked well and the precipitates were significantly enriched for AC091729.7 (Figure 6A). Therefore, the results showed that there is a physical interaction between lncRNA AC091729.7 and SRSF2. Furthermore, Western blot was performed to accurately verify the SRSF2 level in five pairs of SNSCC and adjacent tissues. qRT-PCR was performed to accurately quantify the level of *SRSF2* mRNA in 24 pairs of SNSCC and adjacent tissues. The SRSF2 expression was increased significantly in SNSCC tumors (Figures 6B,C). The downregulation of lncRNA AC091729.7 in RPMI-2650 cells in turn significantly decreased the level of SRSF2 protein (Figure 6D). The SRSF2 mRNA expression in SNSCC tissues was positively correlated with the lncRNA AC091729.7 level (Figure 6E). These findings indicated that AC091729.7 binds to SRSF2. Therefore, follow-up experiments focused on whether the function of AC091729.7 was a result of regulating the SRSF2 expression. In order to investigate whether AC091729.7 promoted the viability of SNSCC cells and wound-healing through SRSF2, we conducted rescue experiments. The overexpression and silencing of SRSF2 were confirmed by Western blot (Figure 7A). Consequently, the overexpression of SRSF2 significantly reduced the cell viability and migration ability induced by AC091729.7 knockdown. Conversely, the silencing of SRSF2 reduced the cell viability and migration ability caused by the overexpression of AC091729.7 (Figures 7B,C). Taken together, the current findings indicated that AC091729.7 regulates SRSF2 to play a carcinogenic role.

## Discussion

Sinonasal carcinoma is a head and neck tumor that occurs in complex anatomical areas of the nasal cavity and paranasal sinuses. SNSCC tumor can commonly invade critical structures such as skull base, eye socket, and brain, thereby declining the rate of survival. The 5-year survival rate was 80% in the early stage patients, which reduced to 30% in the advanced stage patients (1, 22, 23). With the other types of cancer, the molecular pathology of SNSCC was not elucidated sufficiently for early diagnosis (24). Therefore, finding new, sensitive, and specific SNSCC markers is an urgent requirement to improve the early diagnosis, which would have vital clinical significance for improving the prognosis of SNSCC patients. In addition, exploring the underlying pathological mechanisms leading to the pathogenesis of SNSCC and identifying effective therapeutic targets for suppression of the progression is imperative. A previous study reported that high expression of TrkB plays a major role in SNSCC, and TrkB can be used as a potential prognostic indicator for the clinical prognosis (25). Another report identified that p53 overexpression might be related to the occurrence and development of SNSCC (26). A study by Kovarikova et al. (27) suggested that the upregulation of miR-21 was involved in the occurrence and prognosis of sinonasal carcinoma.

The discovery of the role of lncRNA in cancer formation is one of the most important advances in oncology in the last decade. With the rapid progress in sequencing technology, numerous studies have shown that lncRNAs could be used as oncogenes or tumor suppressors during tumorigenesis (28). Like in breast cancer, colon cancer, and Glioblastoma Multiforme, lncRNAs are of great significance (29–31). Several recent studies have reported that multiple lncRNAs are involved in the progression of head and neck cancer; for example, H19, NEAT1, and HOTAIR are overexpressed in laryngeal cancer (32–34). The current study first selected lncRNA AC091729.7 as the research object in SNSCC and found that AC091729.7 expression was upregulated in tumor tissues as assessed by microarray, RNAscope *in situ* hybridization, and qRT-PCR in independent cohorts. The 869-bp length lncRNA, AC091729.7, is newly discovered and localized on chromosome 7. The role of this lncRNA has not been investigated in malignancy. In the current study, the clinical data analysis of the SNSCC cancer patients showed that high level of AC091729.7 significantly correlated with the T grade, recurrence, and poor survival of SNSCC. Furthermore, the knockdown of AC091729.7 could significantly suppress the proliferation and invasion of SNSCC cells both *in vitro* and *in vivo*. These results suggested that AC091729.7 promotes the malignant phenotypes and functions as an oncogene in SNSCC. As a nucleus-enriched lncRNA, the mechanism of AC091729.7 in SNSCC remains unclear. Furthermore, nucleus-localized lncRNAs are emerging as common structures involved in many cellular processes, including epigenetic regulation, chromosomal interactions, and transcriptional regulation (35).

Previous studies indicated that Ser/Arg-rich (SR) proteins could directly participate in the development of tumor suppressors or apoptotic regulators, carcinogenic effect, acting as proto-oncogenes, or regulating splicing, and the activity of proto-oncogenes (36–38). SRSF2 is a member of serine/arginine-rich (SR) protein family and is a critical part of cell structure speckle. The upregulation of SRSF2 protein has been confirmed in many cancers (37–42). The protoarray, RIP, and Western blot findings indicated that SRSF2 was increased and bound to AC091729.7 in SNSCC. Thus, the function of SRSF2 is consistent with the carcinogenic function of lncRNA AC091729.7. Furthermore, the inhibitory effect of AC091729.7 knockdown on the proliferation of SNSCC cells could be rescued by the overexpression of SRSF2. These data indicated that the carcinogenic role of lncRNA AC091729.7 in SNSCC was effectuated via regulation of the SRSF2 protein.

## Conclusions

In the present study, we found that a novel lncRNA, AC091729.7, was prominently overexpressed in SNSCC tissues and associated with the progression of SNSCC patients. Furthermore, the downregulation of AC091729.7 significantly inhibited the ability of proliferation and invasion of SNSCC cells. The oncogenic effects of AC091729.7 are related to the regulation of SRSF2 protein. In summary, AC091729.7 may be a prognostic marker for SNSCC and a potential target for therapeutic intervention.

## Data Availability Statement

The datasets generated for this study can be found in the NCBI Gene Expression Omnibus (GSE138809).

## Ethics Statement

The studies involving human participants were reviewed and approved by the Ethics Committee of Harbin Medical University. The patients/participants provided their written informed consent to participate in this study. The animal study was reviewed and approved by the Ethics Committee of Harbin Medical University.

## Author Contributions

YS, QL, LQ, and BYu designed the study. LQ, QL, ML, and LT developed the methodology, performed the analysis. YS and BYu wrote the manuscript. TW, BYa, and XK collected the data, cultured cell lines. XZ, LY, and YL did PCR, Western blot assay, RIP, Xenografts in mice. BYu did ISH.

### Conflict of Interest

The authors declare that the research was conducted in the absence of any commercial or financial relationships that could be construed as a potential conflict of interest.
