# Corrigendum: A novel LncRNA, AC091729.7 promotes sinonasal squamous cell carcinomas proliferation and invasion through binding SRSF2

**DOI:** 10.3389/fonc.2024.1476128

**Published:** 2024-09-30

**Authors:** Boyu Yu, Linmei Qu, Tianyi Wu, Bingrui Yan, Xuan Kan, Xuehui Zhao, Like Yang, Yushan Li, Ming Liu, Linli Tian, Yanan Sun, Qiuying Li

**Affiliations:** ^1^ Department of Otorhinolaryngology, Head and Neck Surgery, The Second Affiliated Hospital, Harbin Medical University, Harbin, China; ^2^ Department of Otorhinolaryngology, Head and Neck Surgery, The Fifth Affiliated Hospital, Harbin Medical University, Daqing, China; ^3^ Department of Otorhinolaryngology, Head and Neck Surgery, Henan Provincial People’s Hospital, People’s Hospital of Zhengzhou University, Zhengzhou, China

**Keywords:** long non-coding RNA, AC091729.7, sinonasal squamous cell carcinomas, serine/arginine rich splicing factor 2, prognosis

In the published article, there was an error in the legend for **Figure 3F** Transwell invasion assay as published. The corrected **Figure 3** and its legend appear below.

**Figure 3:**
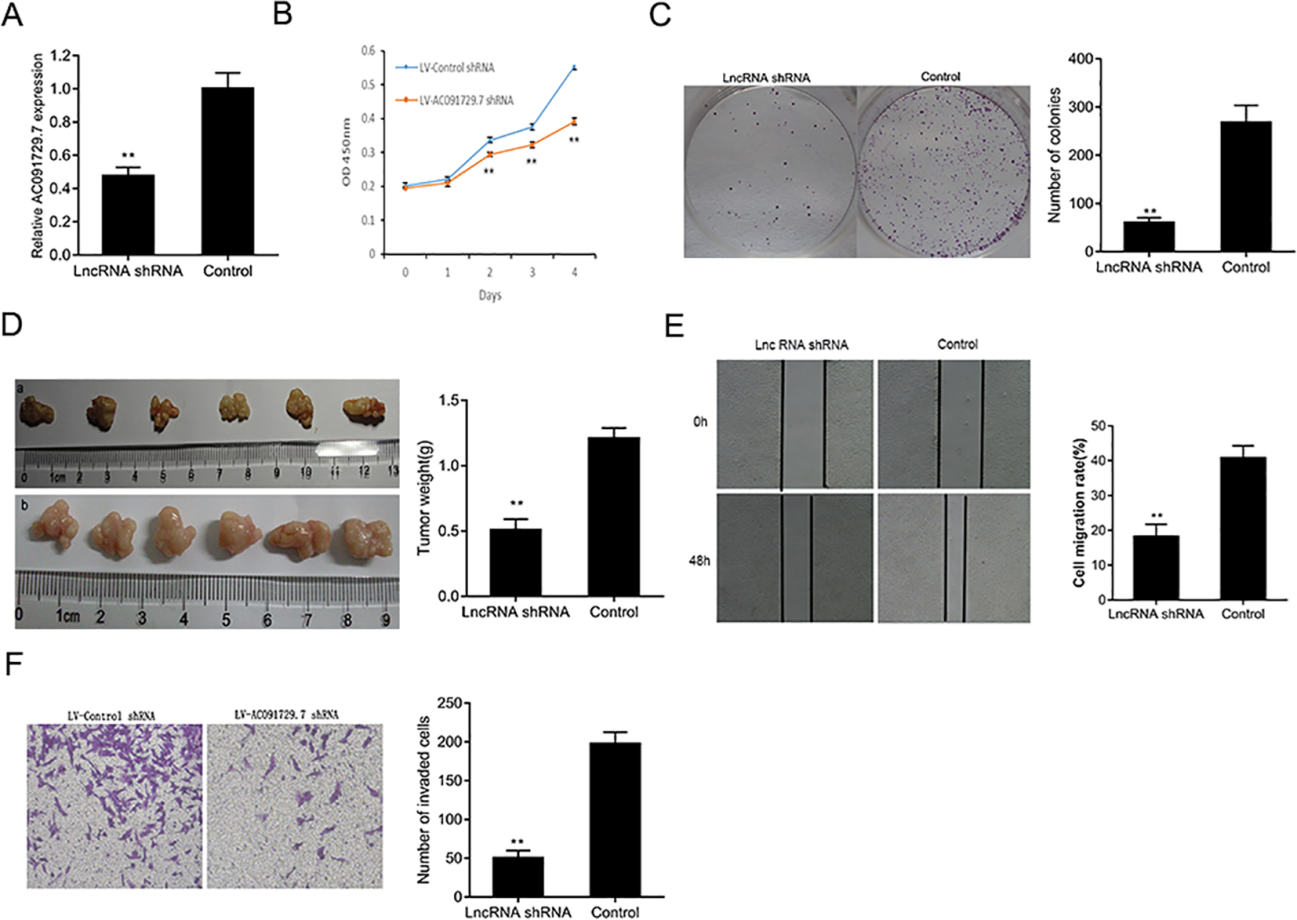
The knockdown of AC091729.7 inhibits the proliferation, migration, and invasion of SNSCC cells. **(A)** Relative RNA level of AC091729.7 was decreased in SNSCC cells with AC091729.7 knockdown. **(B)** CCK-8 showed that the viability of SNSCC cells was inhibited after the downregulation of AC091729.7 expression. **(C)** Colony-formation assay suggested that SNSCC cell proliferation was inhibited after AC091729.7 knockdown. **(D)** Subcutaneous xenograft SNSCC tumors developed in nude mice after RPMI-2650 cells were transfected with lentivirus encoding (a) AC091729.7 shRNA; (b) control shRNA. **(E)** Wound healing cell migration assay. **(F)** Transwell invasion assay (**p < 0.01).

The authors apologize for this error and state that this does not change the scientific conclusions of the article in any way. The original article has been updated.

